# An independent validation of the kidney failure risk equation in an Asian population

**DOI:** 10.1038/s41598-020-69715-3

**Published:** 2020-07-31

**Authors:** Min Woo Kang, Navdeep Tangri, Yong Chul Kim, Jung Nam An, Jeonghwan Lee, Lilin Li, Yun Kyu Oh, Dong Ki Kim, Kwon Wook Joo, Yon Su Kim, Chun Soo Lim, Jung Pyo Lee

**Affiliations:** 1 Department of Internal Medicine, Seoul National University Hospital, Seoul, Korea; 2 Department of Internal Medicine, Rady Faculty of Health Sciences, Max Rady College of Medicine, University of Manitoba, Winnipeg, Canada; 3 Department of Internal Medicine, Hallym University Sacred Heart Hospital, Anyang, Korea; 4 Department of Internal Medicine, Seoul National University Boramae Medical Center, Seoul, Korea; 5 Department of Internal Medicine, Seoul National University College of Medicine, Seoul, Korea; 6 Department of Intensive Care Unit, Yanbian University Hospital, Jilin, China

**Keywords:** Risk factors, End-stage renal disease

## Abstract

Predicting the risk of end-stage renal disease (ESRD) progression facilitates appropriate nephrology care of patients with chronic kidney disease (CKD). Previously, the kidney failure risk equations (KFREs) were developed and validated in several cohorts. The purpose of this study is to validate the KFREs in a Korean population and to recalibrate the equations. A total of 38,905 adult patients, including 13,244 patients with CKD stages G3–G5, who were referred to nephrology were recruited. Using the original KFREs (4-, 6- and 8-variable equations) and recalibration equations, we predicted the risk of 2- and 5-year ESRD progression. All analyses were conducted in CKD stages G3-G5 patients as well as the total population. In CKD stages G3–G5 patients, All the original 4-, 6- and 8-variable equations showed excellent areas under the receiver operating characteristic curve of 0.87 and 0.83 for the 2- and 5-year risk of ESRD, respectively. The results of net reclassification improvement, integrated discrimination index and Brier score showed that recalibration improved the prediction models in some cases. The original KFREs showed high discrimination in both CKD stages G3–G5 patients and the total population referred to nephrology in this large Korean cohort. KFREs can be implemented in Korean health systems and can guide nephrology referrals and other CKD-related treatment decisions.

## Introduction

Globally, the prevalence of chronic kidney disease (CKD) and end-stage renal disease (ESRD) is increasing and becoming challenging to manage from a cost perspective^1–4^. In addition, both CKD and ESRD are known to be associated with high mortality and high risk of cardiovascular disease^5^. Although there are some proven therapies to slow the progression to ESRD, it is difficult to improve the clinical course in advanced disease. For these reasons, aggressive prevention and close monitoring are necessary before CKD progression occurs. Early planning for the initiation of dialysis and transplantation has been advocated. However, treatment and intervention are recommended only for patients at high risk of ESRD progression because of the risks and costs of management^6–10^. Accurate prediction for the risk of CKD progression to ESRD and timely referral to nephrology are important for making proper clinical decisions, but such prediction is difficult because of the variability in rate of progression, severity of kidney disease, and comorbid conditions among patients^11,12^.

Tangri et al. previously developed kidney failure risk equations (KFREs) using demographic and laboratory data to predict the risk of progression of CKD to kidney failure^13^. The risk equations were developed and validated in patients with CKD stages G3–G5 in Canada. Moreover, Tangri et al. validated KFRE in 721,357 patients with CKD stages G3–G5 in 31 multinational cohorts spanning 4 continents^14^. Through this validation, KFREs showed high discrimination and adequate calibration. The purpose of this study was to validate the KFREs in patients who were referred to the nephrology department in Korea.

It is important to refer patients with a high risk of renal failure to nephrology for appropriate management. Early and appropriate referral to nephrology is important because it improves patient outcomes, and estimated glomerular filtration rate (eGFR) is the primary criteria used to guide nephrology referrals in Korea^15–17^. To identify whether the eGFR is better or worse than the KFREs when determining the referral to nephrology, we evaluated the performance of KFREs in the total population followed up at nephrology, including non-CKD patients, and compared KFREs with the eGFR as the predictor of progression to ESRD.

## Results

### Study subjects

**Figure 1:**
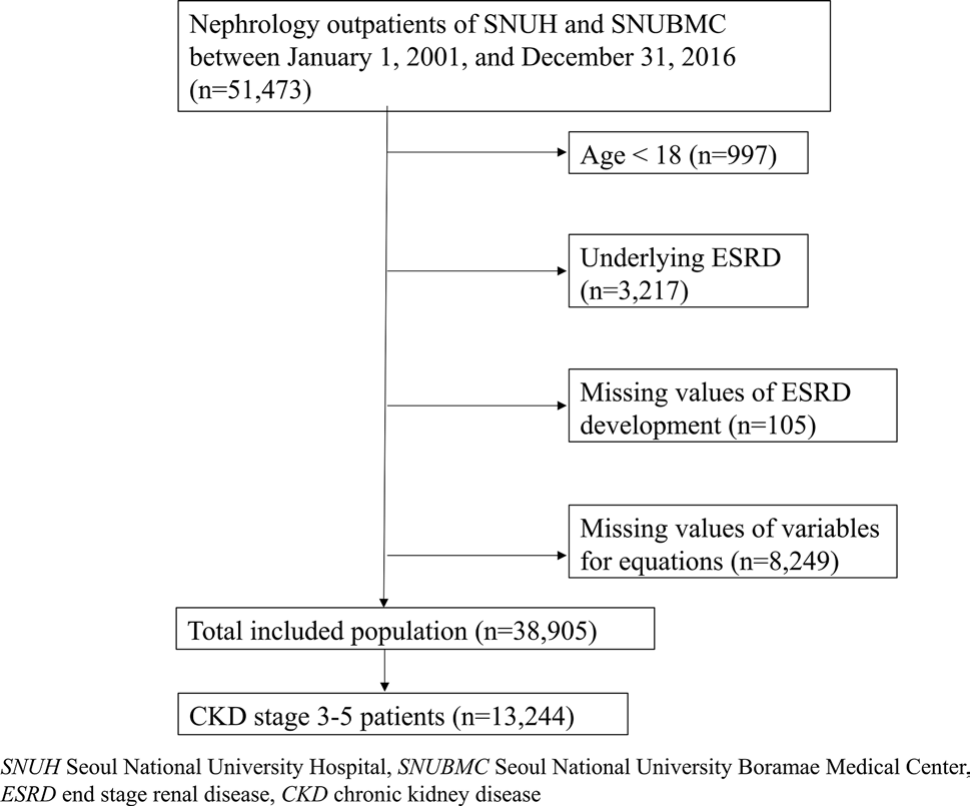
Diagram showing the study population.

With inclusion and exclusion criteria, 38,905 patients were included in the analysis, and the mean follow-up duration was 4.0 years. Among the total study subjects, the number of patients who had underlying CKD stages G3–G5 was 13,244, and the mean follow-up duration was 4.1 years (Fig. 1).

### Baseline characteristics

The baseline characteristics of the total study population and CKD stages G3–G5 patients are shown in Table 1. The mean age of the total study population was 55.8 years, and the mean baseline eGFR was 68.4 mL/min/1.73 m^2^. The mean age of the CKD stages G3–G5 patients was 59.9 years, and the mean baseline eGFR was 36.1 mL/min/1.73 m^2^. A total of 4,488 (11.5%) subjects developed ESRD in the total population, and 3,706 (28.0%) subjects developed ESRD in the CKD stages G3–G5 patient group during the follow-up period. The median values with interquartile range (IQR) of age and eGFR of total population were 54.0 with 23.4 years and 70.9 with 35.8 mL/min/1.73 m^2^, respectively. The median values with IQR of age and eGFR of CKD stages G3–G5 patients were 62.2 with 19.8 years and 36.1 with 30.0 mL/min/1.73 m^2^, respectively.

**Table 1:** Baseline characteristics.

Characteristics	Total population	Patients with CKD stages G3–G5
Age (years)	55.8 ± 16.2	59.9 ± 14.6
Male (%)	49.6	58.0
HTN (%)	30.0	47.4
DM (%)	22.3	29.0
GFR (mL/min/1.73 m^2^)	68.4 ± 30.8	36.1 ± 17.1
Urine albumin-to-creatinine ratio (mg/g)	651.2 ± 11,962.9	992.9 ± 2,325.6
Serum calcium (mg/dL)	9.1 ± 0.6	9.0 ± 0.8
Serum phosphate (mg/dL)	3.7 ± 0.8	3.9 ± 1.1
Serum albumin (g/dL)	4.1 ± 0.5	3.9 ± 0.6
Serum total CO_2_ (mmol/L)	26.1 ± 3.9	24.2 ± 4.5
Development of ESRD (%)	11.5	28.0
Observational time (years)	4.0 ± 4.2	4.1 ± 4.1

*CKD* chronic kidney disease, *HTN* hypertension, *DM* diabetes mellitus, *GFR* glomerular filtration rate, *ESRD* end-stage renal disease.

### Validation of the KFREs

**Figure 2:**
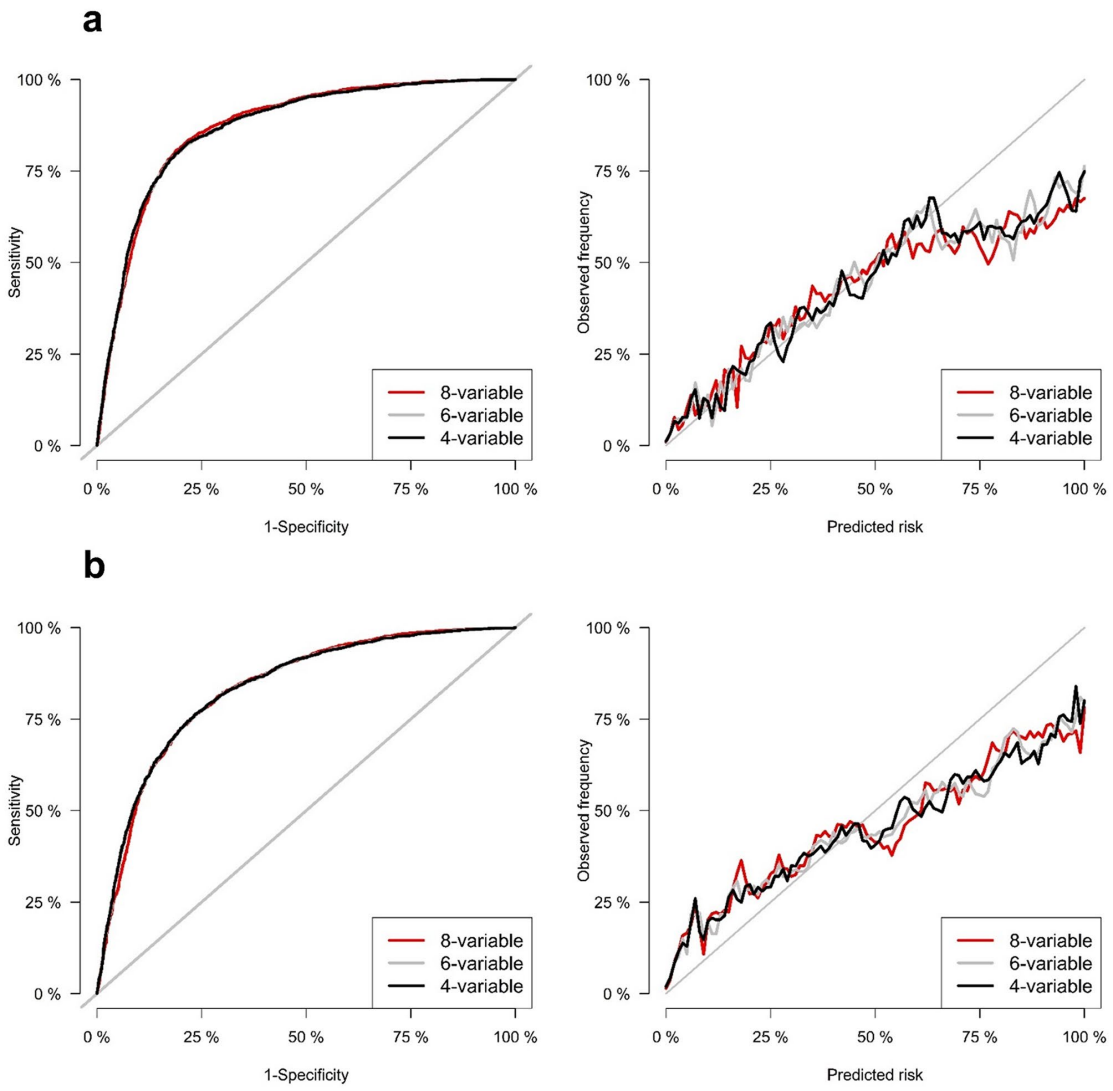
Time-dependent ROC and calibration plots of 2- and 5-year risk prediction models in CKD stages G3-G5 patients. (**a**) 2-year risk prediction. (**b**) 5-year risk prediction.

**Figure 3:**
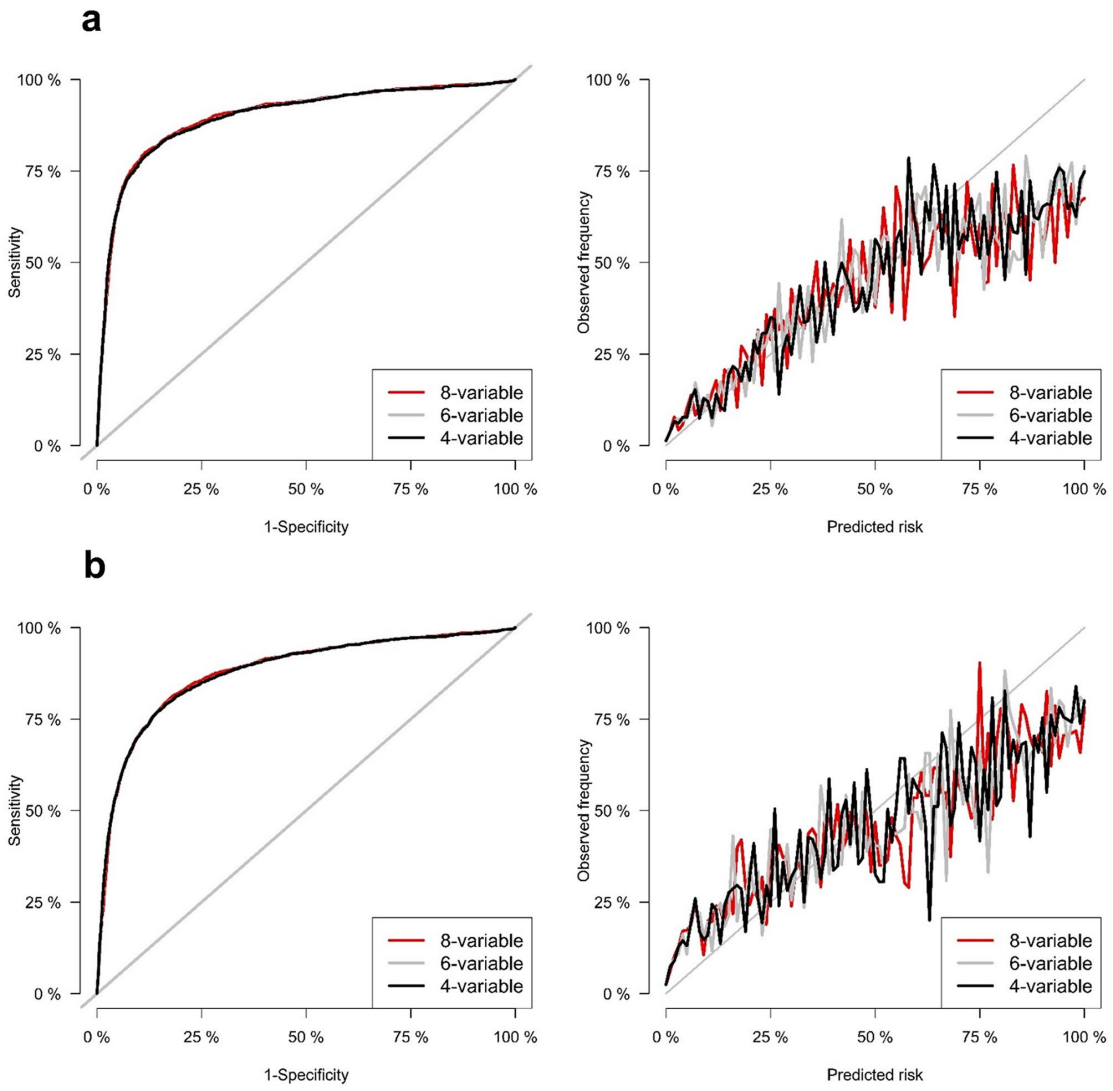
Time-dependent ROC and calibration plots of 2- and 5-year risk prediction models in the total population. (**a**) 2-year risk prediction. (**b**) 5-year risk prediction.

The discrimination of the original 4-, 6-, 8-variable equations was excellent for the 2- and 5-year ESRD risk predictions in both CKD stages G3–G5 patients and the total population. In CKD stages G3-G5 patients, the original 4-, 6-, and 8-variable equations for 2-year ESRD risk showed areas under the time-dependent receiver operating characteristic curve (AUROCs) of 0.868, 0.868, and 0.870, respectively (Table 2). Table 3 showed the results of integrated discrimination index (IDI) and net reclassification improvement (NRI). In the ‘Model’s column in Table 3, the former is the base model and the latter is the new model. For examples, ‘6-variable vs 8-variable’ means that 6-variable is base model and 8-variable is new model. The positive NRI or IDI values mean that the new model is better than the base model. The results of IDI and NRI showed that the 8-variable equation was statistically inferior to 6- and 4-variable equations, while there was no significant difference in 6- and 4-variable equations (Table 3). The original 4-, 6-, and 8-variable equations for 5-year ESRD risk showed AUROCs of 0.834, 0.834, and 0.844, respectively, and the comparison among equations using NRI and IDI showed the same tendency of the 2-year ESRD risk equations (Tables 2, 3). Table 4 showed the results of Brier scores. The lower values of Brier scores mean the better calibrations of models. As a result of comparing the Brier score for evaluating calibration, the 8-variable equation was statistically inferior to the 4- and 6-variable equations for both 2-year and 5-year ESRD risk, while the calibrations of 4- and 6 variable equations were not statistically different (Table 4). Figure 2 shows the time-dependent receiver operating characteristic (ROC) curves and calibration plots for 2- and 5-year ESRD risk in CKD stages G3–G5 patients. In the total population, 4-, 6-, and 8-variable equations for both 2- and 5-year ESRD risk also showed excellent discrimination with high values of AUROC (Supplementary Table S1). The results of NRI, IDI and Brier score for all 2- and 5-year risk equations in the total population showed the same tendency as that in patients with CKD G3-G5 (Supplementary Tables S2, S3). Figure 3 shows the time-dependent ROC curves and calibration plots for 2- and 5-year ESRD risk in the total population.

**Table 2:** Time-dependent AUROCs of the 4-, 6-, and 8-variable equations for predicting 2- and 5-year ESRD development in patients with CKD stages G3–G5.

Model	Original equation AUROC (95% CI)	Recalibration AUROC (95% CI)	P-value
**2-year risk prediction**			
8-variable	0.870 (0.862, 0.879)	0.870 (0.862, 0.879)	0.30
6-variable	0.868 (0.859, 0.877)	0.868 (0.859, 0.877)	0.30
4-variable	0.868 (0.860, 0.877)	0.868 (0.860, 0.877)	0.60
**5-year risk prediction**			
8-variable	0.833 (0.822, 0.843)	0.833 (0.823, 0.843)	0.40
6-variable	0.834 (0.823, 0.844)	0.834 (0.823, 0.844)	0.99
4-variable	0.834 (0.824, 0.845)	0.834 (0.824, 0.845)	0.99

*AUROC* area under the receiver operating characteristic, *ESRD* end-stage renal disease, *CKD* chronic kidney disease.

**Table 3:** Comparing the performances among models for predicting 2- and 5-year ESRD development in patients with CKD stages G3-G5 using IDI and NRI.

Models	IDI (95% CI)	P-value	Continuous NRI (95% CI)	P-value
**2-year risk prediction**				
6-variable vs 8-variable	− 0.016 (− 0.025, − 0.008)	< 0.01	− 0.331 (− 0.364, − 0.272)	< 0.01
4-variable vs 8-variable	− 0.016 (− 0.024, − 0.010)	< 0.01	− 0.330 (− 0.365, − 0.299)	< 0.01
4-variable vs 6-variable	0.000 (− 0.001, 0.002)	0.81	− 0.186 (− 0.281, 0.182)	0.46
**5-year risk prediction**				
6-variable vs 8-variable	− 0.012 (− 0.019, − 0.006)	< 0.01	− 0.317 (− 0.375, − 0.262)	< 0.01
4-variable vs 8-variable	− 0.011 (− 0.017, − 0.006)	< 0.01	− 0.322 (− 0.367, − 0.272)	< 0.01
4-variable vs 6-variable	0.000 (− 0.001, 0.001)	0.75	0.165 (− 0.067, 0.253)	0.24

*ESRD* end-stage renal disease, *CKD* chronic kidney disease, *IDI* Integrated Discrimination Index, *NRI* net reclassification improvement.

**Table 4:** Comparing the performances among models for predicting 2- and 5-year ESRD development in patients with CKD stages G3-G5 using Brier scores.

Model	Brier score (95% CI)	Model comparison	P-value
**2-year risk prediction**			
8-variable	0.116 (0.105, 0.126)	6-variable vs 8-variable	< 0.01
6-variable	0.111 (0.101, 0.122)	4-variable vs 8-variable	< 0.01
4-variable	0.111 (0.101, 0.121)	4-variable vs 6-variable	0.25
**5-year risk prediction**			
8-variable	0.168 (0.152, 0.184)	6-variable vs 8-variable	< 0.01
6-variable	0.165 (0.149, 0.180)	4-variable vs 8-variable	< 0.01
4-variable	0.165 (0.149, 0.180)	4-variable vs 6-variable	0.24

*ESRD* end-stage renal disease, *CKD* chronic kidney disease.

### Comparing predictive performance for ESRD progression of original equations versus recalibration equations

Next, the predictive performance for ESRD progression of recalibration equations was compared with the original equations with the time-dependent AUROC, IDI and NRI analysis. In CKD stages G3–G5 patients, the AUROCs of the recalibration equations were similar to the original equations, and there were no statistically significant differences between recalibration and original equations (Table 2). The results of NRI and IDI showed that the recalibration equations for all 2-year risk and 8-variable 5-year risk equations were superior and that the recalibration equations for 6- and 4-variable 5-year risk equations were inferior to the original equations (Table 5). However, the analysis of Brier score and calibration plot showed the opposite results (Supplementary Fig. S1). Supplementary Table S4 showed the comparisons of Brier scores of the original equations and recalibration equations. The Δ Brier score means the changes of Brier score compared to the original equation after recalibration, and a negative value of Δ Brier score means that the recalibration equation shows the better calibration. The Brier scores of the recalibrated 8- and 6-variable 2-year risk and 8-variable 5-year risk equations were statistically higher, meaning inferior, than those of the original equations and that the Brier scores of the recalibrated 6- and 4-variable 5-year risk equations were statistically lower, meaning superior, than those of the original equations (Supplementary Table S4).

**Table 5:** IDI and NRI (original vs recalibration) for predicting 2- and 5-year ESRD development in patients with CKD stages G3–G5.

Model	IDI (95% CI)	P-value	Continuous NRI (95% CI)	P-value
**2-year risk prediction**				
8-variable	0.025 (0.016, 0.035)	< 0.01	0.317 (0.294, 0.348)	< 0.01
6-variable	0.019 (0.010, 0.027)	< 0.01	0.310 (0.284, 0.334)	< 0.01
4-variable	0.017 (0.006, 0.025)	< 0.01	0.303 (0.272, 0.327)	< 0.01
**5-year risk prediction**				
8-variable	0.007 (0.003, 0.013)	< 0.01	0.155 (0.133, 0.185)	< 0.01
6-variable	− 0.010 (− 0.012, − 0.007)	< 0.01	− 0.251 (− 0.282, − 0.222)	< 0.01
4-variable	− 0.007 (− 0.008, − 0.005)	< 0.01	− 0.245 (− 0.272, − 0.217)	< 0.01

*IDI* Integrated Discrimination Index, *NRI* net reclassification improvement, *ESRD* end-stage renal disease, *CKD* chronic kidney disease.

### Comparing the predictive performance of eGFR and KFREs

Finally, we compared the performance of the original KFREs in the Korean population with the conventional eGFR standard. The eGFR showed time-dependent AUROCs of 0.876 for 2-year ESRD risk prediction and 0.851 for 5-year ESRD risk prediction in the total population. All the original equations for both 2- and 5-year ESRD risk prediction showed statistically better discrimination than eGFR (Table 6). In the total population, the 4-variable equation showed better performance than the 8-variable equation through the results of IDI and NRI, and the results of the Brier score comparison also showed that the 4-variable equation had better calibration. We thought that, among the KFREs, the 4-variable equation was most appropriate for screening of referral to nephrologist because many patients who need the decision of referral to a nephrologist have an eGFR over 60 mL/min/1.73 m^2^, such as the total population of our study cohort. The classification using an eGFR of less than 30 mL/min/1.73 m^2^ showed the same sensitivity of as the 4-variable equation with a threshold > 0.00025% in 5-year risk prediction and threshold > 0.00007% in 2-year risk prediction. However, the 4-variable equation showed higher specificity, positive predictive value and negative predictive value (Supplementary Table S5). We also analyzed the specificity at sensitivities of 0.99, 0.95, 0.90, 0.85, and 0.80 (Supplementary Table S5).

**Table 6:** Time-dependent AUROC of eGFR for predicting 2- and 5-year ESRD development in the total population.

	AUROC (95% CI)	P-value for comparing with original equations		
		4-variable	6-variable	8-variable
2-year risk prediction	0.876 (0.866, 0.885)	< 0.01	< 0.01	< 0.01
5-year risk prediction	0.851 (0.841, 0.860)	< 0.01	0.01	< 0.01

*AUROC* area under the receiver operating characteristic, *eGFR* estimated glomerular filtration rate, *ESRD* end-stage renal disease.

### Analysis using multiple imputation

The number of excluded patients, due to missing values, was 16% of the total population, which was a fairly large proportion. The variables that had missing values were eGFR, urine ACR, serum calcium, serum phosphorus, serum albumin and serum total CO_2_. The other variables, including age, sex, diabetes mellitus and hypertension had no missing values. We compared baseline characteristics, which had no missing values, between the included study population and the excluded population (Supplementary Table S6). There were statistically significant differences of baseline characteristics between the study population and the excluded population. In addition, the results of Cox proportional hazard models for ESRD progression between the excluded population and the study population showed that the included study population had a lower risk of ESRD progression (Supplementary Table S7). Therefore, we used multiple imputation to fill in the missing values and analyzed it in the same way as the study population. After multiple imputation, a total of 16,729 patients with CKD G3–G5 were included in analysis. The time-dependent AUROC values were similar to the AUROC values seen in CKD patients before multiple imputation (Supplementary Table S8). The results of IDI and NRI analysis showed that the 8-variable equation was statistically inferior to the 4- and 6-variable equations, which was the same tendency as the results of the analysis performed on CKD patients before multiple imputation (Supplementary Table S9). As a result of Brier score analysis in the 2-year risk prediction equation, the 8-variable equation showed statistically poor calibration compared to the 4- and 6-variable equations, which is the same tendency in CKD patients before multiple imputation. However, the 8-variable equation for the 5-year risk prediction was not statistically inferior to the 4- and 6-variable equations (Supplementary Table S10). After multiple imputation, the results of NRI showed that the recalibration equations for all 2- and 5-year ESRD risk were better than the original equations (Supplementary Table S11).

## Discussion

In this validation study involving 38,905 subjects, including 13,244 CKD stages G3–G5 patients, the KFREs accurately predicted the 2- and 5-year probabilities of progression to ESRD in Korean patients who initially visited and were referred to the nephrology department. This study demonstrated that the KFREs showed excellent predictive performance not only in CKD stages G3–G5 patients but also in the total population, including patients with normal eGFR. Recalibration using baseline hazards and means of variables in this cohort did not improve discrimination. Recalibration showed improvement of calibration in all equations for 2-year ESRD risk and only in the 8-variable equation for 5-year ESRD risk. To the best of our knowledge, this study was the first to show the superior effect of KFREs for predicting ESRD in a large Asian population with a long-term follow-up period.

The original KFREs reported by Tangri et al. showed excellent predictive performance in the North American and non-North American populations, and they have been validated in several studies^13,14,18–20^. The meta-analysis across 31 cohorts and over 30 countries showed excellent discrimination in predicting 2- and 5-year kidney failure (C statistic 0.90 and 0.88, respectively)^14^. In the KFRE validation study in European CKD patients, the 8-variable equation showed good discrimination (AUROC 0.89) and better performance than the four-variable model (NRI 6.5%) and the three-variable model (NRI 12.4%)^18^.

In the present study, we validated the KFREs in all patients who were followed up in the nephrology department, including subjects with normal eGFR, and found them to show excellent discrimination and good calibration. This total population included patients with CKD stages G1 and G2 whose eGFR was ≧ 60 mL/min/1.73 m^2^. Although their eGFR was normal, many people who initially visited or were referred to nephrology might have early signs of various kidney diseases, such as haematuria and proteinuria. In addition, proteinuria is a strong and independent predictor of ESRD, and haematuria has been suggested to be a risk factor for the progression of CKD^21,22^. Given that the risk of ESRD could also be high in patients with GFR ≧ 60 mL/min/1.73 m^2^, prediction of ESRD development in this population is important. Based on the results of the present study, we suggest that patients with an eGFR ≧ 60 mL/min/1.73 m^2^ who follow up with nephrology could also be evaluated with KFREs. However, since the KFREs were developed in a cohort of CKD stages G3–G5 patients, interpretations of the results of validation in those populations should be performed with caution. Further validation studies of KFREs in subjects with normal eGFR are needed.

This study showed that 8-variable KFREs for both 2- and 5-year ESRD risk were inferior to 4- and 6-variable KFREs in CKD stages G3-G5 patients through the results of IDI, NRI and Brier score. Among 2-year ESRD risk prediction equations, the 8-variable equation had the highest AUROC of 0.870, but the 4- and 6-variable equation had similar AUROC values of 0.868. The meta-analysis reported by Tangri et al. also demonstrated that the discrimination of the 8-variable equation was highest among the 8-, 6- and 4-variable equations^14^. However, the 8-variable equation could be difficult to apply when there are missing values among the 8 variables. Conversely, the 4-variable equation is simpler and can be applied more widely. According to the present study, it is appropriate to use the 4-variable equation for both 2- and 5-year ESRD progression in CKD stages G3–G5 patients.

Current eGFR is known to be associated with the development of ESRD, and the risk of ESRD progression is higher, especially at eGFR < 30 mL/min per 1.73 m^2^^23^. The Kidney Disease: Improving Global Outcomes (KDIGO) guidelines recommend referrals to specialists for patients with GFR < 30 mL/min per 1.73 m^2^^17^. In the present study, the original KFREs showed significantly higher discrimination in predicting 2- and 5-year ESRD risks than eGFR alone in the total population. In addition, the original 4-variable equation with a threshold > 0.00025% in 5-year risk prediction and threshold > 0.00007% in 2-year risk prediction showed higher specificity, positive predictive value (PPV), and negative predictive value (NPV) for ESRD progression than eGFR < 30 mL/min per 1.73 m^2^. Therefore, it could be more reasonable to use the KFREs than eGFR alone in determining nephrology referrals. However, if the analyzed thresholds of equations were used for the decision of referral to nephrologist, many unnecessary patients would be evaluated and treated because it showed too high sensitivity and too low specificity. The values of specificity, PPV and NPV at sensitivities of 0.99, 0.95, 0.90, 0.85, and 0.80 in Supplementary Table S5 could be helpful for clinicians to make decision of referral to nephrologist using 4-variable equation.

In this study, the recalibration equations using the means of variables and baseline hazards were better than the original equation in some cases, and worse in other cases. However, in the CKD patient cohort after multiple imputation, the results of NRI showed that the recalibration equations for all 2- and 5-year ESRD risk were better than the original equations. These findings are particularly important because previous equations in cardiovascular disease, such as the Framingham Study Equations^24,25^, or in kidney disease, such as eGFR estimating equations^26,27^, have required recalibration prior to use in Asian countries. These findings suggest that the KFRE could be applied in the Korean population after recalibrations that substitute the baseline hazard and the mean values of each variable in the original KFRE equations into that of the study cohort. However, the AUROCs of the recalibration equations were almost the same as the AUROCs of the original equations, and the recalibrated 4- and 6-variable equations for 5-year risk in CKD patients before multiple imputation were statistically inferior to the original equations. Moreover, the calibration performances became poor when recalibrated in some equations. Therefore, further study is needed to show that recalibration can improve the performance of equations.

This study has several limitations. First, many people did not have urine albumin-to-creatinine ratio measurements. Although we transformed the urine protein-to-creatinine ratios and urinary dipstick test results into urine albumin-to-creatinine ratio (ACR) as previous studies did, there could be inaccuracies. In addition, as the dipstick test at a single time was converted into the urine ACR, misclassification may occur for transient proteinuria. Second, many subjects had missing values for variables used in the 8-variable equation. Third, since referral patients from only 2 centers of nephrology in Korea were included, validation studies from other centers may be necessary.

In conclusion, the original KFREs showed high discrimination in both CKD stages G3–G5 patients and the total population referred to nephrology when validated in a Korean cohort. KFREs can be more helpful in determining nephrology referrals than eGFR alone. Future studies should evaluate the utility of the KFRE in guiding dialysis access and transplant referral in nephrology practices compared to eGFR or other guideline-based standards of care.

## Method

### Study population

We studied all patients who had visited and followed up at the nephrology clinics of two tertiary hospitals between January 1, 2001, and December 31, 2016. This study cohort is an open cohort and a right censor strategy was used. Patients who initially visited nephrology as outpatients and patients who were referred to nephrology were included. We excluded patients who had underlying ESRD. Patients younger than 18 years of age and patients with missing variables needed for KFRE were excluded. This study was approved by the Institutional Review Board of Seoul National University Hospital. (No. 1910-110-1071) The Institutional Review Board of Seoul National University Hospital waived the need for informed consent due to the study’s retrospective design. All clinical investigations were conducted in accordance with the guidelines of the 2013 Declaration of Helsinki.

First, as in the original KFRE study^13,14^, CKD stages G3-G5 patients whose eGFR was less than 60 mL/min/1.73 m^2^ were selected for this study. In addition, all patients who visited the nephrology outpatient clinic and complied with the inclusion and exclusion criteria were included in the analysis (Fig. 1).

### Variables and development of recalibrated KFREs in the Korean population

There were 4 KFREs developed in the original study: the 3-variable (age, sex, and eGFR), the 4-variable (3-variable + urine ACR), the 6-variable (4-variables + diabetes mellitus and hypertension), and the 8-variable (4-variables + calcium, phosphate, bicarbonate, and albumin) equations (Table 7)^13^. The 4-, 6- and 8-variable equations were validated in a previous meta-analysis and showed high discrimination^14^. In the present study, the 4-, 6-, and 8-variable equations were validated. We recalibrated the equations, replacing the 5- and 2-year ESRD survival rates and the mean values of each variable in the equations with the survival rates and values from the cohort of the present study (eAppendix 1). The validation of recalibration equations was also conducted.

**Table 7:** Original kidney failure risk equations.

Models	Equation
**2-year risk prediction**	
8-variable	$1 - 0.9780^{{{\exp}( - 0.1992 \times ({\text{age}}/10 - 7.036) + 0.1602 \times ({\text{male}} - 0.5642) - 0.4919 \times ({\text{eGFR}}/5 - 7.222) + 0.3364 \times ({\text{logACR}} - 5.137) - 0.3441 \times ({\text{albumin}} - 3.997) + 0.2604 \times ({\text{phosphorous}} - 3.916) - 0.07354 \times ({\text{TCO}}2 - 25.57) - 0.2228 \times ({\text{calcium}} - 9.355))}}$
6-variable	${1 }{-} \, 0.{975}0^{{{\exp}( - 0.{2218 } \times \, ({\text{age}}/{1}0 \, {-}{ 7}.0{36}) + \, 0.{2553 } \times \, \left( {{\text{male }}{-} \, 0.{5642}} \right) \, {-} \, 0.{5541 } \times \, \left( {{\text{eGFR}}/{5 }{-}{ 7}.{222}} \right) \, + \, 0.{4562 } \times \, \left( {{\text{logACR }}{-}{ 5}.{137}} \right) \, {-} \, 0.{1475 } \times \, \left( {{\text{DM }} - \, 0.{51}0{6}} \right) \, + \, 0.{1426 } \times \, \left( {{\text{HTN }}{-} \, 0.{85}0{1}} \right))}}$
4-variable	${1 }{-} \, 0.{975}0^{{{\exp}( - 0.{22}0{1 } \times \, ({\text{age}}/{1}0 \, {-}{ 7}.0{36}) + \, 0.{2467 } \times \, \left( {{\text{male }}{-} \, 0.{5642}} \right) \, {-} \, 0.{5567 } \times \, \left( {{\text{eGFR}}/{5 }{-}{ 7}.{222}} \right) \, + \, 0.{451}0 \, \times \, \left( {{\text{logACR }}{-}{ 5}.{137}} \right))}}$
**5-year risk prediction**	
8-variable	${1 }{-} \, 0.{93}0{1}^{{{\exp}( - 0.{1992 } \times \, \left( {{\text{age}}/{1}0 \, {-}{ 7}.0{36}} \right) \, + \, 0.{16}0{2 } \times \, \left( {{\text{male }}{-} \, 0.{5642}} \right) \, {-} \, 0.{4919 } \times \, \left( {{\text{eGFR}}/{5 }{-}{ 7}.{222}} \right) \, + \, 0.{3364 } \times \, \left( {{\text{logACR }}{-}{ 5}.{137}} \right) \, {-} \, 0.{3441 } \times \, \left( {{\text{albumin }}{-}{ 3}.{997}} \right) \, + \, 0.{26}0{4 } \times \, \left( {{\text{phosphorous }}{-}{ 3}.{916}} \right) \, {-} \, 0.0{7354 } \times \, \left( {{\text{bicarbonate }}{-}{ 25}.{57}} \right) \, {-} \, 0.{2228 } \times \, ({\text{calcium }}{-}{ 9}.{355}))}}$
6-variable	${1 }{-} \, 0.{924}0^{{{\exp}( - 0.{2218 } \times \, \left( {{\text{age}}/{1}0 \, {-}{ 7}.0{36}} \right) \, + \, 0.{2553 } \times \, \left( {{\text{male }}{-} \, 0.{5642}} \right) \, {-} \, 0.{5541 } \times \, \left( {{\text{eGFR}}/{5 }{-}{ 7}.{222}} \right) \, + \, 0.{4562 } \times \, \left( {{\text{logACR }}{-}{ 5}.{137}} \right) \, {-} \, 0.{1475 } \times \, \left( {{\text{DM }}{-} \, 0.{51}0{6}} \right) \, + \, 0.{1426 } \times \, ({\text{HTN }}{-} \, 0.{85}0{1}))}}$
4-variable	${1} - 0.{924}0^{{{\exp}( - 0.{22}0{1 } \times \, \left( {{\text{age}}/{1}0 \, {-}{ 7}.0{36}} \right) \, + \, 0.{2467 } \times \, \left( {{\text{male }}{-} \, 0.{5642}} \right) \, {-} \, 0.{5567 } \times \, \left( {{\text{eGFR}}/{5 }{-}{ 7}.{222}} \right) \, + \, 0.{451}0 \, \times \, ({\text{logACR }}{-}{ 5}.{137}))}}$

*ACR* urine albumin-to-creatinine ratio, *eGFR* estimated glomerular filtration rate, *DM* diabetes mellitus, *HTN* hypertension.

A total of 10 predictor variables, including age, sex, eGFR, urine ACR, serum calcium, serum phosphorus, serum albumin, serum total CO_2_, diabetes mellitus, and hypertension, were obtained to calculate the KFREs. Because bicarbonate is not checked routinely, we used the total CO_2_ value as a bicarbonate value. All variables needed for the KFREs were obtained at baseline from nephrology outpatients. Estimated GFR was calculated using the Modification of Diet in Renal Disease Study (MDRD) Equations^28^. The baseline laboratory value was defined as the first test result within 30 days of the initial nephrology outpatient visit. Comorbidities, which were diabetes mellitus and hypertension, were categorized as present or absent at the time of the initial nephrology outpatient visit. As in the original KFRE study, urine ACR was log transformed. For subjects who had no data on urine ACR, urine protein-to-creatinine ratios were converted to urine ACR by dividing by 2.655 for men and 1.7566 for women, as in the KFRE validation studies^14,29^. For subjects who had no data on either urine ACR or urine protein-to-creatinine ratio, urinary dipsticks were converted to urine ACR (negative as 9, trace as 43, “+” as 81, “++” as 315, and “> ++” as 1,073 mg/g)^14,30,31^. ESRD was defined as the commencement of dialysis or undergoing kidney transplantation.

### Statistical analysis

Statistical analyses were performed using R software (Version 3.6.2. R Core Team (2019). R: A language and environment for statistical computing. R Foundation for Statistical Computing, Vienna, Austria. URL https://www.R-project.org/). Cox proportional hazards models were fit using the variables included in each of the original equations, and we analyzed the baseline hazard. As in previous studies, the KFREs were validated in CKD stages G3–G5 patients. However, all patients who visited the nephrology outpatient clinics were also analyzed for validation. We developed recalibration equations by substituting the baseline hazard and the mean values of each variable in the original KFRE equations into that of our cohort.

Discrimination of the original and recalibrated KFREs was assessed using the time-dependent AUROC. The IDI and Continuous NRI were used to evaluate the prediction performances among the 4-, 6- and 8-variable equations^32–34^. All analyses were conducted in CKD stages G3–G5 patients as well as the total study population. Using the Brier score and calibration plot, the calibration among the 4-, 6- and 8-variable equation models was evaluated and compared^35^.

The predictive performance of eGFR for the ESRD progression in the total population was analyzed using time-dependent AUROC and compared with the AUROC of the original 8-variable equation for prediction of ESRD progression in the total population. The IDI and Continuous NRI were also used to comparison. We calculated the sensitivity to ESRD progression for eGFR thresholds of 30 mL/min/1.73 m^2^ and identified the cut-off value of the 4-variable equation that had the same sensitivity to ESRD progression. We then compared the specificity, PPV, and NPV of the 4-variable equation using the identified cut-off value with that of eGFR thresholds of 30 mL/min/1.73 m^2^. P values < 0.05 were considered significant.

## Supplementary information

Supplementary Information.
