# Supplementary material for: An independent validation of the kidney failure risk equation in an Asian population
Source: Sci Rep. 2020 Jul 31;10:12920. doi: 10.1038/s41598-020-69715-3 (PMC7395750; doi:10.1038/s41598-020-69715-3)
Supplement: Supplementary file 1 — Supplementary Information. [file 41598_2020_69715_MOESM1_ESM.docx]

**An independent validation of the kidney failure risk equation in an Asian population**

Min Woo Kang^1^, Navdeep Tangri^2^, Yong Chul Kim^1^, Jung Nam An^3^, Jeonghwan Lee^4^, Lilin Li^5,6^, Yun Kyu Oh^4,5^, Dong Ki Kim^1,5^, Kwon Wook Joo^1,5^, Yon Su Kim^1,5^, Chun Soo Lim^4,5^, Jung Pyo Lee^4,5^

^1^Department of Internal Medicine-Nephrology, Seoul National University Hospital, Korea, Republic of

^2^Section of Nephrology, Department of Internal Medicine, Max Rady College of Medicine, Rady Faculty of Health Sciences, University of Manitoba, Winnipeg, Canada

^3^Department of Internal Medicine, Hallym University Sacred Heart Hospital, Anyang, Korea
^4^Department of Internal Medicine-Nephrology, Seoul National University Boramae Medical Center, Korea, Republic of

^5^Department of Internal Medicine, Seoul National University College of Medicine, Seoul, Korea

^6^Department of Intensive Care Unit, Yanbian University Hospital, Jilin, China

*Correspondence: Jung Pyo Lee, MD, PhD

Associate Professor

Department of Internal Medicine, Seoul National University College of Medicine and Seoul National University Boramae Medical Center

20, Boramae-ro 5-gil, Dongjak-gu, Seoul, 07061, Korea

Tel. +82-2-870-3206; Fax +82-2-870-2826; E-mail: nephrolee@gmail.com

**Running title:** Validation of KFRE in an Asian population

eAppendix 1. Equations to apply 2 or 5-year risk in the 4-, 6-, or 8-variable Kidney Failure Risk Equations in patients with CKD stages G3-G5 and the total population

4-variable equation, Patient 2-year risk:

| Original | 1 – 0.9750 ^ exp (-0.2201 × (age/10 – 7.036) + 0.2467 × (male – 0.5642) – 0.5567 × (eGFR/5 – 7.222) + 0.4510 × (logACR – 5.137)) |
| --- | --- |
| Recalibration in patients with CKD stages G3-G5 | 1 – 0.8851 ^ exp (-0.2201 × (age/10 – 5.986) + 0.2467 × (male – 0.5804) – 0.5567 × (eGFR/5 – 7.2265) + 0.4510 × (logACR – 5.428)) |
| Recalibration in the total population | 1 – 0.9811 ^ exp (-0.2201 × (age/10 – 5.403) + 0.2467 × (male – 0.4962) – 0.5567 × (eGFR/5 – 13.6886) + 0.4510 × (logACR – 4.396)) |

4-variable equation, Patient 5-year risk:

| Original | 1 – 0.9240 ^ exp (-0.2201 × (age/10 – 7.036) + 0.2467 × (male – 0.5642) – 0.5567 × (eGFR/5 – 7.222) + 0.4510 × (logACR – 5.137)) |
| --- | --- |
| Recalibration in patients with CKD stages G3-G5 | 1 – 0.7668 ^ exp (-0.2201 × (age/10 – 5.986) + 0.2467 × (male – 0.5804) – 0.5567 × (eGFR/5 – 7.2265) + 0.4510 × (logACR – 5.428)) |
| Recalibration in the total population | 1 – 0.9611 ^ exp (-0.2201 × (age/10 – 5.403) + 0.2467 × (male – 0.4962) – 0.5567 × (eGFR/5 – 13.6886) + 0.4510 × (logACR – 4.396)) |

6-variable equation, Patient 2-year risk:

| Original | 1 – 0.9750 ^ exp (-0.2218 × (age/10 – 7.036) + 0.2553 × (male – 0.5642) – 0.5541 × (eGFR/5 – 7.222) + 0.4562 × (logACR – 5.137) – 0.1475 × (DM – 0.5106) + 0.1426 × (HTN – 0.8501)) |
| --- | --- |
| Recalibration in patients with CKD stages G3-G5 | 1 – 0.8927 ^ exp (-0.2218 × (age/10 – 5.986) + 0.2553 × (male – 0.5804) – 0.5541 × (eGFR/5 – 7.2265) + 0.4562 × (logACR – 5.428) – 0.1475 × (DM – 0.2859) + 0.1426 × (HTN – 0.4738)) |
| Recalibration in the total population | 1 – 0.9827 ^ exp (-0.2218 × (age/10 – 5.403) + 0.2553 × (male – 0.4962) – 0.5541 × (eGFR/5 – 13.6886) + 0.4562 × (logACR – 4.396) – 0.1475 × (DM – 0.2232) + 0.1426 × (HTN – 0.2999)) |

6-variable equation, Patient 5-year risk:

| Original | 1 – 0.9240 ^ exp (-0.2218 × (age/10 – 7.036) + 0.2553 × (male – 0.5804) – 0.5541 × (eGFR/5 – 7.2265) + 0.4562 × (logACR – 5.428) – 0.1475 × (DM – 0.5106) + 0.1426 × (HTN – 0.8501)) |
| --- | --- |
| Recalibration in patients with CKD stages G3-G5 | 1 – 0.7842 ^ exp (-0.2218 × (age/10 – 5.986) + 0.2553 × (male – 0.5793) – 0.5541 × (eGFR/5 – 7.2958) + 0.4562 × (logACR – 5.413) – 0.1475 × (DM – 0.2859) + 0.1426 × (HTN – 0.4738)) |
| Recalibration in the total population | 1 – 0.9648 ^ exp (-0.2218 × (age/10 – 5.403) + 0.2553 × (male – 0.4962) – 0.5541 × (eGFR/5 – 13.6886) + 0.4562 × (logACR – 4.396) – 0.1475 × (DM – 0.2232) + 0.1426 × (HTN – 0.2999)) |

8-variable equation, Patient 2-year risk:

| Original | 1 – 0.9780 ^ exp (-0.1992 × (age/10 – 7.036) + 0.1602 × (male – 0.5642) – 0.4919 × (eGFR/5 – 7.222) + 0.3364 × (logACR – 5.137) – 0.3441 × (albumin – 3.997) + 0.2604 × (phosphorous – 3.916) – 0.07354 × (TCO2 – 25.57) – 0.2228 × (calcium – 9.355)) |
| --- | --- |
| Recalibration in patients with CKD stages G3-G5 | 1 – 0.8836 ^ exp (-0.1992 × (age/10 – 5.986) + 0.1602 × (male – 0.5804) – 0.4919 × (eGFR/5 – 7.2265) + 0.3364 × (logACR – 5.428) – 0.3441 × (albumin – 3.886) + 0.2604 × (phosphorous – 3.908) – 0.07354 × (TCO2 – 24.22) – 0.2228 × (calcium – 8.955)) |
| Recalibration in the total population | 1 – 0.9800 ^ exp (-0.1992 × (age/10 – 5.403) + 0.1602 × (male – 0.4962) – 0.4919 × (eGFR/5 – 13.6886) + 0.3364 × (logACR – 4.396) – 0.3441 × (albumin – 4.06) + 0.2604 × (phosphorous – 3.689) – 0.07354 × (TCO2 – 26.07) – 0.2228 × (calcium – 9.124)) |

8-variable equation, Patient 5-year risk:

| Original | 1 – 0.9301 ^ exp (-0.1992 × (age/10 – 7.036) + 0.1602 × (male – 0.5642) – 0.4919 × (eGFR/5 – 7.222) + 0.3364 × (logACR – 5.137) – 0.3441 × (albumin – 3.997) + 0.2604 × (phosphorous – 3.916) – 0.07354 × (bicarbonate – 25.57) – 0.2228 × (calcium – 9.355)) |
| --- | --- |
| Recalibration in patients with CKD stages G3-G5 | 1 – 0.7637 ^ exp (-0.1992 × (age/10 – 5.986) + 0.1602 × (male – 0.5804) – 0.4919 × (eGFR/5 – 7.2265) + 0.3364 × (logACR – 5.428) – 0.3441 × (albumin – 3.886) + 0.2604 × (phosphorous – 3.908) – 0.07354 × (TCO2 – 24.22) – 0.2228 × (calcium – 8.955)) |
| Recalibration in the total population | 1 – 0.9577 ^ exp (-0.1992 × (age/10 – 5.403) + 0.1602 × (male – 0.4962) – 0.4919 × (eGFR/5 – 13.6886) + 0.3364 × (logACR – 4.396) – 0.3441 × (albumin – 4.06) + 0.2604 × (phosphorous – 3.689) – 0.07354 × (TCO2 – 26.07) – 0.2228 × (calcium – 9.124)) |

Figure S1. Calibration plots of original and recalibration equations in CKD stages G3-G5 patients. **a**. 2-year risk prediction. **b**. 5-year risk prediction


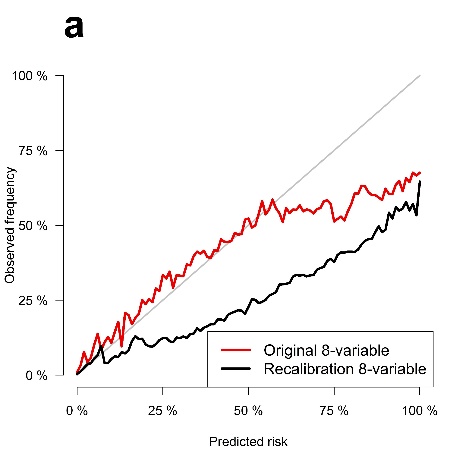

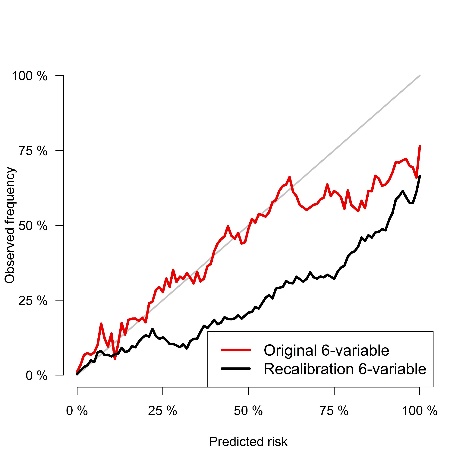

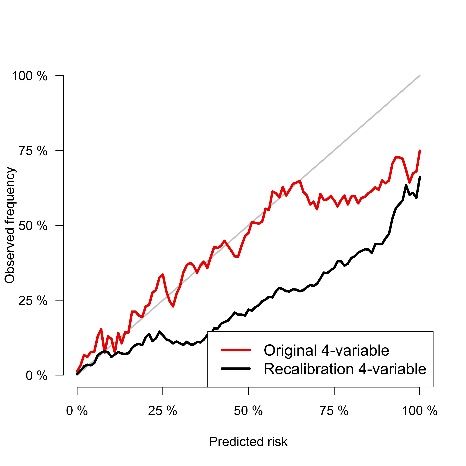

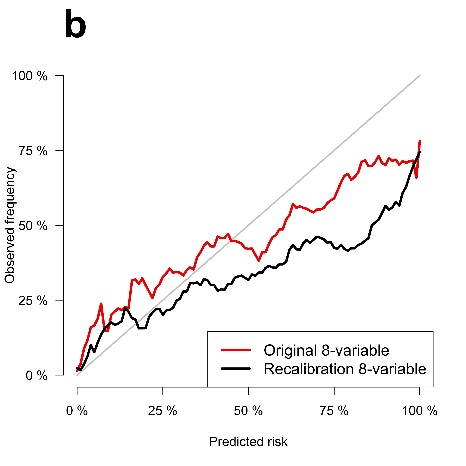

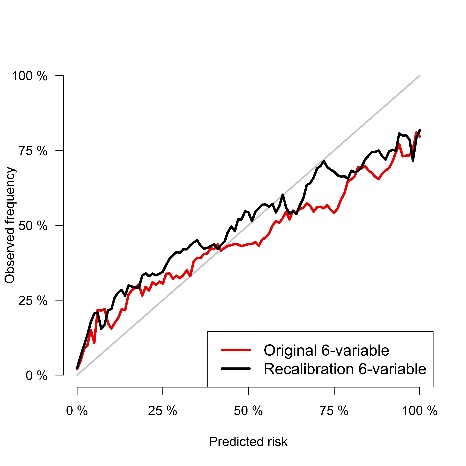

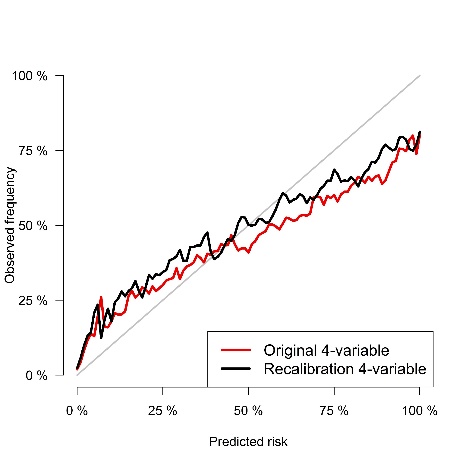


Table S1. Time-dependent AUROCs of 4-, 6-, and 8-variable equations for predicting 2- and 5-year ESRD development in the total population

| Model | Original equation AUROC  (95% CI) | Recalibration AUROC  (95% CI) | p-value |
| --- | --- | --- | --- |
| 2-year risk prediction |  |  |  |
| 8-variable | 0.899 (0.891, 0.907) | 0.899 (0.891, 0.907) | 0.4 |
| 6-variable | 0.897 (0.889, 0.905) | 0.897 (0.889, 0.905) | 0.06 |
| 4-variable | 0.896 (0.887, 0.904) | 0.895 (0.887, 0.904) | 0.02 |
| 5-year risk prediction |  |  |  |
| 8-variable | 0.879 (0.871, 0.887) | 0.879 (0.871, 0.887) | 0.05 |
| 6-variable | 0.878 (0.870, 0.886) | 0.877 (0.869, 0.885) | 0.06 |
| 4-variable | 0.878 (0.870, 0.886) | 0.877 (0.869, 0.885) | 0.04 |

*AUROC: area under the receiver operating characteristic, ESRD: end-stage renal disease

Table S2. Comparing the performances among models for predicting 2- and 5-year ESRD development in the total population using IDI and NRI

| Models | IDI (95% CI) | p-value | Continuous NRI (95% CI) | p-value |
| --- | --- | --- | --- | --- |
| 2-year risk prediction |  |  |  |  |
| 6-variable vs 8-variable | -0.014 (-0.021, -0.006) | <0.01 | -0.387 (-0.411, -0.352) | <0.01 |
| 4-variable vs 8-variable | -0.013 (-0.022, -0.006) | <0.01 | -0.387 (-0.417, -0.360) | <0.01 |
| 4-variable vs 6-variable | 0.000 (-0.001,0.001) | 0.99 | -0.366 (-0.405, 0.273) | 0.24 |
| 5-year risk prediction |  |  |  |  |
| 6-variable vs 8-variable | -0.009 (-0.014, -0.005) | <0.01 | -0.413 (-0.439, -0.372) | <0.01 |
| 4-variable vs 8-variable | -0.009 (-0.014, -0.005) | <0.01 | -0.414 (-0.435, -0.380) | <0.01 |
| 4-variable vs 6-variable | 0.000 (-0.001,0.001) | 0.85 | -0.126 (-0.292, 0.328) | 0.77 |

* ESRD: end-stage renal disease, IDI: Integrated Discrimination Index, NRI: net reclassification improvement

Table S3. Comparing the performances among models for predicting 2- and 5-year ESRD development in the total population using Brier scores

| Model | Brier score (95% CI) | Model comparison | p-value |
| --- | --- | --- | --- |
| 2-year risk prediction |  |  |  |
| 8-variable | 0.052 (0.038, 0.066) | 6-variable vs 8-variable | <0.01 |
| 6-variable | 0.050 (0.036, 0.064) | 4-variable vs 8-variable | <0.01 |
| 4-variable | 0.050 (0.036, 0.064) | 4-variable vs 6-variable | 0.31 |
| 5-year risk prediction |  |  |  |
| 8-variable | 0.084 (0.061, 0.107) | 6-variable vs 8-variable | 0.02 |
| 6-variable | 0.083 (0.060, 0.105) | 4-variable vs 8-variable | <0.01 |
| 4-variable | 0.083 (0.060, 0.105) | 4-variable vs 6-variable | 0.28 |

* ESRD: end-stage renal disease

Table S4. Comparing the calibration of the original and recalibration equations for predicting 2- and 5-year ESRD development in patients with CKD stages G3-G5 using Brier scores

| Recalibration models | Brier score (95% CI) | Δ Brier score (95% CI) | p-value |
| --- | --- | --- | --- |
| 2-year risk prediction |  |  |  |
| 8-variable | 0.151 (0.141, 0.161) | 0.035 (0.030, 0.040) | <0.01 |
| 6-variable | 0.149 (0.139, 0.158) | 0.038 (0.033, 0.042) | <0.01 |
| 4-variable | 0.152 (0.142, 0.161) | 0.041 (0.036, 0.046) | 0.31 |
| 5-year risk prediction |  |  |  |
| 8-variable | 0.186 (0.170, 0.201) | 0.017 (0.013, 0.022) | <0.01 |
| 6-variable | 0.162 (0.146, 0.178) | -0.003 (-0.005, -0.001) | <0.01 |
| 4-variable | 0.162 (0.146, 0.178) | -0.002 (-0.004, -0.001) | <0.01 |

* ESRD: end-stage renal disease, CKD: chronic kidney disease

Table S5. Sensitivity, specificity, PPV and NPV of the 4-variable original equation and eGFR in the total population

|  | Sensitivity | Specificity | Positive predictive value | Negative predictive value |
| --- | --- | --- | --- | --- |
| 2-year risk prediction |  |  |  |  |
| eGFR <30 mL/min/1.73 m^2^ | 0.995 | 0.007 | 0.076 | 0.943 |
| 4-variable equation >0.00007% | 0.995 | 0.016 | 0.076 | 0.974 |
| 4-variable equation >0.031% | 0.950 | 0.446 | 0.123 | 0.991 |
| 4-variable equation >0.23% | 0.900 | 0.691 | 0.192 | 0.988 |
| 4-variable equation >1.17% | 0.850 | 0.812 | 0.270 | 0.985 |
| 4-variable equation >4.00% | 0.800 | 0.876 | 0.344 | 0.982 |
| 5-year risk prediction |  |  |  |  |
| eGFR <30 mL/min/1.73 m^2^ | 0.996 | 0.007 | 0.135 | 0.904 |
| 4-variable equation >0.00025% | 0.996 | 0.014 | 0.136 | 0.955 |
| 4-variable equation >0.075% | 0.950 | 0.410 | 0.201 | 0.981 |
| 4-variable equation >0.35% | 0.900 | 0.630 | 0.275 | 0.976 |
| 4-variable equation >1.11% | 0.850 | 0.750 | 0.347 | 0.970 |
| 4-variable equation >3.00% | 0.800 | 0.823 | 0.413 | 0.964 |

*PPV: positive predictive value, NPV: negative predictive value, eGFR: estimated glomerular filtration rate

Table S6. Comparison of baseline characteristics between included and excluded populations

| Characteristics | Study population (n=38,905) | Excluded population (n=8,249) | p-value |
| --- | --- | --- | --- |
| Age (years) | 55.8±16.2 | 56.7±15.9 | <0.01 |
| Male (%) | 49.6 | 43.8 | <0.01 |
| HTN (%) | 30.0 | 37.9 | <0.01 |
| DM (%) | 22.3 | 15.8 | <0.01 |
| ESRD progression (%) | 11.5 | 15.4 | <0.01 |
| Mortality (%) | 12.5 | 17.5 | <0.01 |

*HTN: hypertension, DM: diabetes mellitus, ESRD: end-stage renal disease

Table S7. Hazard ratios of ESRD progression and mortality for the study population compared with the excluded population using Cox proportional hazard models

|  | Unadjusted HR (95% CI) | p-value | Adjusted HR (95% CI) | p-value |
| --- | --- | --- | --- | --- |
| ESRD progression | 0.86 (0.81-0.91) | <0.01 | 0.87 (0.82-0.93) | <0.01 |
| Mortality | 0.59 (0.56-0.63) | <0.01 | 0.66 (0.62-0.70) | <0.01 |

* ESRD: end-stage renal disease, HR: hazard ratio

** Adjusted variables: Age, sex, hypertension, diabetes mellitus

Table S8. Time dependent AUROCs of 4-, 6-, and 8-variable equations for predicting 2- and 5-year ESRD development in patients with CKD stages G3-G5 after multiple imputation

| Model | Original equation AUROC  (95% CI) | Recalibration AUROC  (95% CI) | p-value |
| --- | --- | --- | --- |
| 2-year risk prediction |  |  |  |
| 8-variable | 0.860 (0.853, 0.868) | 0.860 (0.853, 0.868) | 0.20 |
| 6-variable | 0.856 (0.849, 0.864) | 0.856 (0.849, 0.864) | 0.30 |
| 4-variable | 0.857 (0.849, 0.865) | 0.857 (0.849, 0.865) | 0.30 |
| 5-year risk prediction |  |  |  |
| 8-variable | 0.824 (0.815, 0.834) | 0.824 (0.815, 0.834) | 0.80 |
| 6-variable | 0.821 (0.812, 0.831) | 0.821 (0.812, 0.831) | 0.50 |
| 4-variable | 0.822 (0.813, 0.832) | 0.822 (0.813, 0.832) | 0.50 |

* AUROC: area under the receiver operating characteristic, ESRD: end-stage renal disease, CKD: chronic kidney disease

Table S9. Comparing the performances among models for predicting 2- and 5-year ESRD development in patients with CKD stages G3-G5 after multiple imputation using IDI and NRI

| Models | IDI (95% CI) | p-value | Continuous NRI (95% CI) | p-value |
| --- | --- | --- | --- | --- |
| 2-year risk prediction |  |  |  |  |
| 6-variable vs 8-variable | -0.008 (-0.014, -0.001) | <0.01 | -0.286 (-0.322, -0.061) | <0.01 |
| 4-variable vs 8-variable | -0.008 (-0.013, -0.001) | <0.01 | -0.295 (-0.324, -0.066) | <0.01 |
| 4-variable vs 6-variable | 0.000 (-0.001,0.001) | 0.55 | -0.246 (-0.291, 0.089) | 0.18 |
| 5-year risk prediction |  |  |  |  |
| 6-variable vs 8-variable | -0.003 (-0.011, 0.000) | <0.01 | -0.194 (-0.281, -0.017) | <0.01 |
| 4-variable vs 8-variable | -0.004 (-0.009, -0.000) | <0.01 | -0.210 (-0.274, -0.035) | <0.01 |
| 4-variable vs 6-variable | -0.001 (-0.001,0.000) | <0.01 | -0.020 (-0.091, 0.128) | 0.73 |

* ESRD: end-stage renal disease, CKD: chronic kidney disease, IDI: Integrated Discrimination Index, NRI: net reclassification improvement

Table S10. Comparing the performances among models for predicting 2- and 5-year ESRD development in patients with CKD stages G3-G5 after multiple imputation using Brier scores

| Model | Brier score (95% CI) | Model comparison | p-value |
| --- | --- | --- | --- |
| 2-year risk prediction |  |  |  |
| 8-variable | 0.123 (0.112, 0.134) | 6-variable vs 8-variable | <0.01 |
| 6-variable | 0.119 (0.107, 0.130) | 4-variable vs 8-variable | <0.01 |
| 4-variable | 0.118 (0.107, 0.129) | 4-variable vs 6-variable | 0.12 |
| 5-year risk prediction |  |  |  |
| 8-variable | 0.177 (0.161, 0.193) | 6-variable vs 8-variable | 0.18 |
| 6-variable | 0.176 (0.160, 0.191) | 4-variable vs 8-variable | 0.06 |
| 4-variable | 0.175 (0.160, 0.191) | 4-variable vs 6-variable | <0.01 |

* ESRD: end-stage renal disease, CKD: chronic kidney disease

Table S11. IDI and NRI (original vs recalibration) for predicting 2- and 5-year ESRD development in patients with CKD stages G3-G5 after multiple imputation

| Model | IDI (95% CI) | p-value | Continuous NRI (95% CI) | p-value |
| --- | --- | --- | --- | --- |
| 2-year risk prediction |  |  |  |  |
| 8-variable | 0.028 (0.020, 0.034) | <0.01 | 0.313 (0.294, 0.337) | <0.01 |
| 6-variable | 0.023 (0.015, 0.030) | <0.01 | 0.310 (0.285, 0.331) | <0.01 |
| 4-variable | 0.022 (0.014, 0.031) | <0.01 | 0.307 (0.274, 0.336) | <0.01 |
| 5-year risk prediction |  |  |  |  |
| 8-variable | 0.011 (0.004, 0.014) | <0.01 | 0.158 (0.133, 0.181) | <0.01 |
| 6-variable | 0.005 (-0.001, 0.010) | 0.10 | 0.145 (0.119, 0.170) | <0.01 |
| 4-variable | 0.004 (-0.001, 0.009) | 0.28 | 0.136 (0.111, 0.160) | <0.01 |

*IDI: Integrated Discrimination Index, NRI: net reclassification improvement, ESRD: end-stage renal disease, CKD: chronic kidney disease
